# Validity and reliability of the Structured Clinical Interview for the Trauma and Loss Spectrum (SCI-TALS)

**DOI:** 10.1186/1745-0179-4-2

**Published:** 2008-01-28

**Authors:** Liliana Dell'Osso, M Katherine Shear, Claudia Carmassi, Paola Rucci, Jack D Maser, Ellen Frank, Jean Endicott, Liliana Lorettu, Carlo A Altamura, Bernardo Carpiniello, Francesco Perris, Ciro Conversano, Antonio Ciapparelli, Marina Carlini, Nannina Sarno, Giovanni B Cassano

**Affiliations:** 1 Department of Psychiatry, Neurobiology, Pharmacology and Biotechnologies, University of Pisa, Italy; 2 Columbia University, New York, USA; 3 Western Psychiatric Institute and Clinic, University of Pittsburgh, USA; 4 Department of Psychiatry, University of California, San Diego, USA; 5 Clinic of Psychiatry, University of Sassari, Italy; 6 Department of Psychiatry, University of Milan, Italy; 7 Department of Public Health-Section of Psychiatry, University of Cagliari, Italy; 8 Department of Psychiatry, University of Naples, Italy

## Abstract

**Background:**

DSM-IV identifies three stress response disorders (acute stress (ASD), post-traumatic stress (PTSD) and adjustment disorders (AD)) that derive from specific life events. An additional condition of complicated grief (CG), well described in the literature, is triggered by bereavement.

**Methods:**

This paper reports on the reliability and validity of the Structured Clinical Interview for Trauma and Loss Spectrum (SCI-TALS) developed to assess the spectrum of stress response. The instrument is based on a spectrum model that emphasizes soft signs, low-grade symptoms, subthreshold syndromes, as well as temperamental and personality traits comprising clinical and subsyndromal manifestations. Study participants, enrolled at 6 Italian Departments of Psychiatry, included consecutive patients with PTSD (N = 48), CG (N = 44), and controls (N = 48).

**Results:**

We showed good reliability and validity of the SCI-TALS. Domain scores were significantly higher in participants with PTSD or CG compared to controls. There were high correlations between specific SCI-TALS domains and corresponding scores on established measures of similar constructs. Participants endorsing grief and loss events reported similar scores on all instruments, except those with CG who scored significantly higher on the domain of grief reactions.

**Conclusion:**

These results support the existence of a specific grief-related condition and the proposal that different forms of stress response have similar manifestations.

## Introduction

The Structured Clinical Interview for Trauma and Loss Spectrum (SCI-TALS) is an assessment instrument designed to quantify clinical features associated with the experience of stressful life events. DSM-IV [1] categorizes stress-related disorders as either acute stress disorder (ASD), post-traumatic stress disorder (PTSD) or adjustment disorder (AD). Both ASD and PTSD are specifically linked to highly dangerous and/or life-threatening experiences. By contrast, AD is defined by a range of low-level symptoms that occur in response to any life stress and persist for only a limited time. AD is widely regarded as a problematic diagnosis. Horowitz [2] proposed AD to be defined as a stress response syndrome, grouping it with the stress-related disorders in DSM.

There are no criteria in DSM-IV [1] for a syndrome specifically linked to difficult losses, yet a growing body of evidence strongly supports the occurrence of a bereavement-related syndrome of complicated grief (CG) [3-6]. Intrusions, avoidance and failure to adapt also characterize this condition. Horowitz [2] further suggested that complicated grief could be considered a stress response syndrome. Given this way of thinking, stressful life events can be broadly categorized as those that entail experience of a threatening negative life event and those that entail loss of an important positive relationship or situation. We took this perspective in developing the SCI-TALS and designed it to include a spectrum of negative life events and losses and to assess responses that include intrusion, avoidance, emotionality and failure to adapt.

The SCI-TALS is one of a series of "spectrum" instruments that comprise a set of structured clinical interviews and self report questionnaires that evaluate the lifetime occurrence of isolated criterion and non-criterion symptoms, behavioral tendencies and temperament-like traits associated with a given DSM-IV disorder. In the spectrum model, subthreshold or atypical manifestations occur before, during or after onset of a full-blown DSM-IV disorder. Spectrum manifestations may comprise early onset precursors, prodromal features of onset or recurrence, or persistent residual symptoms, which interfere with overall functioning and quality of life. The extent of lifetime exposure to loss and threat events may comprise a risk factor for vulnerability to illness following an important life event. Lifetime symptom burden and/or existence of certain behavioral traits may contribute to the degree of impairment associated with a DSM-IV disorder. We believe that spectrum assessment can be useful for a range of clinical purposes, such as treatment selection, treatment outcome assessment, follow-up, and identifying subthreshold comorbidity [7-10]. We have developed and validated instruments for most of the DSM-IV disorders [11-18].

The purpose of this paper is to describe the SCI-TALS and to document its acceptability, reliability and validity. Data for the present report were collected between May 2004 and December 2005. The Ethics Committee of the Azienda Ospedaliera Universitaria of Pisa approved all recruitment and assessment procedures. Eligible subjects provided written informed consent, after receiving a complete description of the study and having the opportunity to ask questions. Subjects were not paid for their participation in accordance to the Italian laws for clinical studies.

## Methods

### Study Participants

A consecutive group of 140 study participants was recruited, including 92 outpatients and inpatients, presenting for treatment at one of 6 Italian Departments of Psychiatry (Pisa, Cagliari, Milano, Napoli, Sassari and Siena), and 48 healthy controls. Eligible patients met DSM-IV-TR criteria for PTSD (n = 48) [1] or complicated grief, determined by a score of at least 25 on the Inventory of Complicated Grief (n = 44) [19]. Healthy controls (n = 48) were individuals without any history of psychiatric disorders presenting at the Departments of Ophthalmology of the local Universities for a routine sight control, and their friends and relatives. Individuals with severe medical illness, neurological diseases, substance abuse or psychotic symptoms in the month preceding the index assessment, or inability to participate because of the severity of psychiatric symptoms were excluded.

### Instruments and assessments

The Structured Clinical Interview for DSM-IV axis-I disorders (SCID-I/P) [20] was used to determine DSM-IV diagnosis by psychiatrists trained and certified in the use of the study instruments at the Department of Psychiatry of the University of Pisa.

Participants completed the Inventory of Complicated Grief (ICG, translated into Italian by Dr. C. Carmassi and Dr. A. Fagiolini in March 2004) [19] to determine the presence of complicated grief, defined by a score of 25 or higher. The Italian translation was back-translated, revised and translated again.

The Impact of Event Scale (IES) [21] is a widely used scale that has excellent psychometric properties. This instrument assesses intrusion and avoidance symptoms that characterize stress response syndromes.

The Structured Clinical Interview for Trauma and Loss Spectrum [see Additional file 1] was developed by the authors, who comprise the Italian-American team of researchers belonging to the spectrum project. Originally developed in English, the interview was then translated into Italian, back translated, and revised for inconsistencies between the two languages. In the present study we used the final Italian version.

The SCI-TALS includes 116 items exploring the lifetime experience of a range of loss and/or traumatic events and lifetime symptoms, behaviors and personal characteristics that might represent manifestations and/or risk factors for the development of a stress response syndrome. The instrument is organized into 9 domains. Items responses are coded in a dichotomous way (yes/no) and domain scores are obtained by counting the number of positive answers.

Domain I (Items 1–10) catalogues a range of loss events, including death of a loved one, loss of an important relationship, loss of property, losses of physical functioning, or loss of social and economical status. Several authors have pointed to the fact that low magnitude events (e.g.: divorce, serious illness and financial reverses) may produce typical symptoms of traumatic stress, i.e. intrusion, avoidance and arousal [22-25]. In line with these studies, and with the spectrum concept, we included a range of severity of loss and trauma (Domain III) events as possible inciting events.

Domain II (Items 11–37) describes grief reactions that include a range of typical, atypical and sub-syndromal symptoms, related to the possible occurrence of complicated grief, conceptualized as a loss-specific stress response disorder. Loss specific items include longing, yearning and searching for the lost person or place, daydreams about what was lost, a need to reminisce, spending time with objects that are associated with the lost person or place, and frequent intense pangs of grief and sadness related to the loss. Stress response items include intrusions of recurrent upsetting images, avoidance of reminders of the loss, and failure to adapt (difficulty accepting the death, guilt or remorse, feeling life has no purpose and impairment in functioning). This domain also includes a section with 7 items targeting trait-like interpersonal functioning that might comprise a risk factor for persistent grief. Examples include the need to be a caregiver, difficulty asking for help, and sensitivity to separation from loved ones.

Domain III (Items 38–58) lists events that range from DSM-IV qualifying traumas (e.g.: combat, natural disasters, sexual abuse, severe accidents) and "low-magnitude" events (e.g.: failures at school or at work, sexual harassment, abortion), that the patient might have experienced in his/her lifetime. Domain IV (Items 59–76) includes a range of emotional, physical and cognitive responses to loss and/or traumatic events identified in Domains I and III. Domain V (Items 77–85), Domain VI (Items 86–97) and Domain VIII (Items 106–110) include re-experiencing, avoidance and numbing, and arousal symptoms respectively. Domain VII (Items 98–105) targets maladaptive coping, and a last Domain (IX; Items 111–116) includes an experimental list of 6 personality traits that are not included in the analyses.

We evaluated the acceptability of the SCI-TALS using questions asking whether the interview was interesting, reassuring, distressing, and helpful for a better understanding of the disorder for either the patient or the physician. Items were rated on a 0–3 scale, where 0 = not at all, 1 = a little, 2 = much, 3 = very much.

### Statistical analyses

We examined reliability of the SCI-TALS by analyzing correlations between domains and internal consistency of domains. Kuder-Richardson coefficient, a variant of the alpha coefficient for dichotomous items [26], was used to determine the internal consistency. Test-retest and inter-rater reliability of the SCI-TALS was performed at Pisa site only, by having different raters conduct a second SCI-TALS evaluation within 14 days of the initial assessment. We examined reliability using the infraclass correlation coefficient. Shrout criteria [27] were used to define the range of reliability: 0–0.10 virtually none, 0.11–0.40 slight, 0.41–0.60 fair, 0.61–0.80 moderate, 0.81–1 substantial.

We examined the validity of the SCI-TALS by comparing mean domain scores among subjects with PTSD, CG and controls, using one-way analysis of variance followed by post-hoc comparisons with Dunnett's C-test, that allows for heterogeneity of variance between groups. The alpha level was corrected for multiple comparisons (0.016 = 0.05/3). Analysis of covariance was performed to control for potential confounders such as age and gender. Comparisons of categorical variables across groups were conducted by using the 2 × 3 chi-square tests, followed by 2 × 2 chi-square tests. Data analyses were carried out using SPSS 12.0.1.

## Results

### Demographic and Clinical Characteristics of Study Participants

The study group characteristics are provided in Table 1. Controls were younger than CG patients. The majority of patients with CG (84.1%) and of controls (64.6%) were females, while patients with PTSD were equally distributed by gender. Patients with CG were more likely to be widowed than the other groups, had a lower educational level and were less likely to be employed than controls. As expected, CG patients had a significantly higher ICG total score compared to PTSD and controls (Table 1). Fifty percent of individuals with CG also met DSM-IV criteria for PTSD. Also as expected, both CG and PTSD participants scored higher on the IES than controls. Of the 48 PTSD participants, 48% met criteria for lifetime or current major depression, 33% for panic disorder, 6.3% for generalized anxiety disorder, 6.3% for obsessive-compulsive disorder, 4.2% for bipolar II disorder, and 3% for other disorders (including eating disorders or substance use disorder). Of the 44 CG participants, 75% met criteria for lifetime or current major depression, 22.7% for panic disorder, 9.1% for generalized anxiety disorder, 13.6% for bipolar II disorder, 5% for other disorders (including alcohol use disorder, eating disorders or social anxiety disorder).

**Table 1:** Demographic and clinical characteristics of the study samples

	PTSD N = 48	CG N = 44	Controls N = 48	Test, significance (p)
	Mean ± S.D.	Mean ± S.D.	Mean ± S.D.	

*Age*	44.8 ± 14.3	49.3 ± 14.5	41.2 ± 12.2	F = 4.0, p < 0.05, CG>C
	*N (%)*	*N (%)*	*N (%)*	
*Female*	25 (51.2)	37 (84.1)	31 (64.6)	Chi-square = 10.6, p < 0.01, CG>PTSD
*Marital status*				Chi-square = 27.8, p < 0.001
*Single*	14 (29.2)	12 (27.3)	23 (47.9)	
*Married/living with partner*	32 (66.7)	18 (40.9)	24 (50.0)	
*Widows-ers*	1 (2.1)	12 (27.3)	1 (2.1)	
*>8 y. of education*	32 (66.7)	24 (54.5)	38 (79.2)	Chi-square = 6.3, p < 0.05, C>CG
*Employed full/part time*	30 (62.6)	21 (47.7)	53 (72.9)	Chi-square = 6.2, p = 0.05, C>CG
	Mean ± S.D.	Mean ± S.D.	Mean ± S.D.	
IES Total score	26.1 ± 17.2	30.3 ± 15.0	12.0 ± 11.8	F = 19.7, p < 0.001; CG, PTSD>C
Intrusive	13.4 ± 8.9	16.0 ± 8.5	6.6 ± 6.6	F = 16.8, p < 0.001; CG, PTSD>C
Avoidance	12.8 ± 9.4	14.4 ± 8.5	5.4 ± 5.7	F = 16.8, p < 0.001, CG, PTSD>C
ICG Total score	9.3 ± 7.1	38.8 ± 10.5	5.1 ± 6.5	F = 215.8, p < 0.001, CG>PTSD, C

### Acceptability of the SCI-TALS

The SCI-TALS was administered to patients and controls in one session of approximately 30 minutes. No one refused to participate and no participants failed to complete the interview. The acceptability of the interview was excellent: 96.9% of participants rated it as much or very much interesting, and nearly half (49.6%) rated it as much or very much reassuring, 51.8% and 82.5% found that it was helpful to better understand the patients' own problems and to provide useful information to the physician, respectively. However, 32.8% of patients found the interview much or very much distressing.

### Reliability of the SCI-TALS

We examined the internal consistency of the domains and correlations of domains in the pooled sample of individuals with either PTSD or CG diagnosis (Table 2). All Kuder-Richardson coefficients exceeded the minimum standard of 0.50 suggested for group comparison by Helmstadter [28]. Additionally, all domains and 12 out of 16 sub-domains exceeded the 0.70 standard for individual comparisons suggested by Nunnally [29]. For domains I, III and IX the internal consistency was not determined because these were checklists of events or of personality characteristics rather than symptoms. As reported in Table 3, correlations between domains were all positive and significant, with Pearson's r ranging between 0.46 and 0.76 (p < 0.01). Test-retest/inter-rater reliability was excellent, with infraclass correlation coefficients values exceeding .90 for each of the domains (Table 4).

**Table 2:** Internal consistency (Kuder-Richardson coefficients) of the SCI-TALS Domains

*DOMAINS*	*#ITEMS*	*KR-20*
I – Loss events	10	/
II – Grief reactions	27	0.916
III – Potentially traumatic events	21	/
IV – Reaction to losses or upsetting events	18	0.863
V – Re-experiencing	9	0.809
VI – Avoidance and Numbing	11	0.858
VII – Maladaptive coping	8	0.773
VIII – Arousal	6	0.789
IX – Personal Characteristics-Risk Factors	7	/

**Table 3:** Pearson's correlations between SCI-TALS Domains

*Domains*	*IV Reaction to losses or upsetting events*	*V Re-experiencing*	*VI Avoidance and Numbing*	*VII Maladaptive coping*	*VIII Arousal*
*II – Grief reactions*	0.549**	0.538**	0.545**	0.468**	0.521**
*IV – Reaction to losses or upsetting events*		0.709**	0.710**	0.535**	0.722**
*V – Re-experiencing*			0.762**	0.528**	0.706**
*VI – Avoidance and Numbing*				0.541**	0.676**
*VII – Maladaptive coping*					0.567**

** p < 0.01

**Table 4:** Test-retest and inter-rater reliability.

DOMAINS	INTRACLASS CORRELATION COEFFICIENT
I – Loss events	.975
II – Grief reactions	.992
III – Potentially traumatic events	.974
IV – Reaction to losses or upsetting events	.981
V – Re-experiencing	.975
VI – Avoidance and Numbing	.995
VII – Maladaptive coping	.993
VIII – Arousal	.972
IX – Personal Characteristics-Risk Factors	.969

### Validity of SCI-TALS

Mean SCI-TALS domain scores for each of the groups are provided in Table 5, together with the results of the one-way ANOVA and the post-hoc pair wise comparisons. Not surprisingly, all participants reported experiencing similar lifetime rates of loss. Controls reported a mean of 3 lifetime losses, PTSD patients just under 4 and CG patients a mean of 4. Given that CG patients were the oldest group, these slight differences are likely not meaningful. However differences in the response to loss are significant and meaningful. CG participants reported significantly greater levels (almost double) of grief reactions than either PTSD patients or controls. Interestingly, PTSD patients also reported grief reactions that were almost twice as great as controls. Also of note, the two patient groups did not differ on the remaining 6 Domains (Table 2), both scored significantly higher than controls on all Domains. Participants with CG endorsed fewer traumatic events than PTSD patients and more than controls. However CG patients' scores were not different from those with PTSD on any of the stress response domains.

**Table 5:** SCI-TALS Domain total scores in the study samples

DOMAINS	PTSD N = 48	CG N = 44	Controls N = 48	F	*p*	*Dunnett post Hoc comparison at p = 0.016*
	Mean ± S.D.	Mean ± S.D.	Mean ± S.D.			

I – Loss events	3.77 ± 1.87	4.02 ± 1.84	2.83 ± 1.26	6.52	<.01	CG > controls
II – Grief reactions	6.42 ± 5.13	12.00 ± 3.80	3.23 ± 3.21	52.63	<.001	CG> PTSD > controls
III – Potentially traumatic events	5.06 ± 3.07	3.91 ± 2.86	2.25 ± 1.85	13.75	<.001	PTSD> CG > controls
IV – Reaction to losses or upsetting events	10.27 ± 3.50	10.14 ± 3.22	3.62 ± 3.04	64.30	<.001	PTSD, CG > controls
V – Re-experiencing	5.10 ± 2.35	5.14 ± 2.19	1.17 ± 1.36	60.91	<.001	PTSD, CG > controls
VI – Avoidance and numbing	5.89 ± 2.88	5.70 ± 2.72	0.92 ± 1.35	64.97	<.001	PTSD, CG > controls
VII – Maladaptive coping	1.44 ± 1.76	1.93 ± 1.73	0.17 ± 0.43	18.68	<.001	PTSD, CG > controls
VIII – Arousal	3.25 ± 1.39	3.36 ± 1.51	0.81 ± 1.12	54.07	<.001	PTSD, CG > controls

Analysis of covariance was performed on the SCI-TALS domains to determine whether the differences between groups depended on gender and age imbalance. No association was found between age and the SCI-TALS domains. Gender was associated with the domains 'grief reactions', 'maladaptive copying' and 'arousal', but this association did not affect the differences among diagnostic groups.

## Discussion

This paper introduces the Structured Clinical Interview for Trauma and Loss Spectrum (SCI-TALS), a new spectrum instrument focused on assessment of trauma and loss-related experiences, acute event reactions and persistent symptoms and behavioral tendencies that occur in association with these stressful events. Results provide evidence for the reliability and validity of the SCI-TALS when administered to patients with post-traumatic stress disorder, complicated grief and normal controls. We found excellent inter-rater reliability of this interview when administered by different raters a mean of 2 weeks apart. As expected, patients who met DSM-IV criteria for PTSD and ICG criteria for complicated grief scored significantly higher than a comparison control group. This supports the validity of this instrument as a measure of trauma-loss spectrum.

We further believe that our results provide support for grouping stress response syndromes that describe responses to a range of difficult life events [30,31]. Such a grouping would include current DSM-IV diagnoses of ASD, PTSD and AD, as well as complicated grief. We did not include patients with DSM-IV adjustment or acute stress disorders in this study. However, results on both SCI-TAL and IES support the appropriateness of grouping CG and PTSD.

As far as we know, this is the first paper to directly compare individuals diagnoses with PTSD and those diagnosed with CG on the IES and other symptoms characteristic of the two disorders. Our results support the idea that there are strong commonalities in these two conditions. For example, scores on the IES are virtually identical. Prior studies of CG have shown, similar elevations on the IES [5,6,32]. Our results also indicate that CG and PTSD differ importantly with respect to key CG symptoms such as yearning and longing and sadness.

We also draw attention to the observation that nearly a third of the patients who participated in this study found the interview distressing. It is widely known that asking patients to talk about trauma and bereavement can be distressing. Although all still agreed to continue the interview, this reaction was not seen in our other spectrum projects, and should be noted. Clinicians conducting assessments of traumatized and bereaved individuals need to be cognizant of their sensitivity to activation around discussion of these issues.

We believe that this interview, like the other spectrum instruments we have developed, has the advantage of helping patients understand themselves and feel understood by their clinician. Moreover, lifetime spectrum symptoms of a range of mood and anxiety disorders were found to contribute to impairment [33,34], to represent a risk factor for suicidality [35], and to influence treatment outcome [9,36,37].

We believe the spectrum approach provides a more specific description of the clinical features of each patient with potentially important implications for treatment choice and research. We also think that a less restrictive approach to the definition of the potentially traumatic events, than that defined in DSM-IV, would particularly help clinicians to explore more accurately post-traumatic stress conditions. Moreover, the evidence of a different profile for those suffering the consequences of trauma versus loss suggests that the spectrum approach might help identify specific phenotypes to be used in clinical, neurobiological and genetic studies. Further studies are warranted to assess the potential utility of this approach in clinical practice.

## Conclusion

The present study documented the validity and reliability of a new spectrum assessment instrument, the Structured Clinical Interview for Trauma and Loss Spectrum (SCI-TALS). Domain scores were significantly higher in participants with PTSD or CG compared to controls and high correlations between specific SCI-TALS domains and corresponding scores on established measures of similar constructs were reported. Participants endorsing grief and loss events reported similar scores on all instruments, except those with CG who scored significantly higher on the domain of grief reactions, supporting the existence of a specific grief-related condition and the proposal that different forms of stress response have similar manifestations.

## Competing interests

The author(s) declare that they have no competing interests.

## Authors' contributions

LDO, KS, CCarm, PR, JDM, EF, JE and GBC participated to the SCI-TALS elaboration and development as part of the "Spectrum Project" research group. LDO, KS and CCarm conceived the study and participated in its design. LDO and CCarm coordinated the Multicenter national validation study from the Department of Psychiatry of the University of Pisa. CCarm, AC, NS, CConv and MC collected data from all patients recruited at the University of Pisa site. LL, CAA, BC, FP coordinated the data collection form the Universities of Sassari, Milan, Cagliari and Naples respectively. LDO, MKS and CCarm drafted the manuscript. PR participated in the design of the study and performed the statistical analysis. All authors read and approved the final manuscript.

## Supplementary Material

Additional file 1 Structured Clinical Interview for Trauma and Loss Spectrum (SCI-TALS). The full text of the Structured Clinical Interview for Trauma and Loss Spectrum (SCI-TALS) is provided. Click here for file
