# Supplementary material for: Validity and reliability of the Structured Clinical Interview for the Trauma and Loss Spectrum (SCI-TALS)
Source: Clin Pract Epidemiol Ment Health. 2008 Jan 28;4:2. doi: 10.1186/1745-0179-4-2 (PMC2265706; doi:10.1186/1745-0179-4-2)
Supplement: Additional file 1 — Structured Clinical Interview for Trauma and Loss Spectrum (SCI-TALS). The full text of the Structured Clinical Interview for Trauma and Loss Spectrum (SCI-TALS) is provided. [file 1745-0179-4-2-S1.doc]

**Additional file 1:**

**Structured Clinical Interview for Trauma and Loss Spectrum (SCI-TALS)**

Introduction:

Thank you for coming in to talk with me today. The interview we are going to do is focused on losses and upsetting events that you may or may not have experienced in your life and your reactions to them. We want to know whether you had these reactions at any time, even if it was a long time ago. There are eight sections of the interview and it should take us about half an hour to complete it. Do you have any questions before we start?

Interviewer: I am going to begin by asking you about some losses that you may have experienced.

# Domain I. LOSS EVENTS

Did any of the following things ever happen to you?

|  | …a change in homes, caregivers, schools, jobs, etc. that you didn’t want or regretted? | Yes | No |
| --- | --- | --- | --- |
|  | …separation from a close friend, romantic partner, or family member because of relocation, hospitalization, military service, or because of an argument or disagreement? | Yes | No |
|  | …a painful break-up with a romantic partner or a close friend? | Yes | No |
|  | …a divorce in your family? | Yes | No |
|  | …the loss or death of a cherished pet? | Yes | No |
|  | …being neglected or abandoned? | Yes | No |
|  | …the death of a close friend or relative? | Yes | No |
|  | …a miscarriage, stillbirth, or abortion? *Note to interviewer: can apply to male subjects as well as female.* | Yes | No |

|  | Did you lose your sight, hearing, or have a serious disability? | Yes | No |
| --- | --- | --- | --- |
|  | Did you have any other important losses, whether people, places or things that I haven’t asked you about? What were they?  *Record here:* | Yes | No |

*Note to interviewer: If the subject has not endorsed any losses up to this point, skip to Domain III (Traumatic Events).*

# Domain II. GRIEF REACTIONS

# Since you experienced these losses, have you ever had a period of time when…

|  | …you had a lot of trouble accepting the loss? | Yes | No |
| --- | --- | --- | --- |
|  | …you constantly longed for the way things used to be? | Yes | No |
|  | …you longed or searched for a loved one or a familiar place in a way that seemed excessive and/or uncontrollable? | Yes | No |
|  | …you daydreamed a lot about the person or thing you lost? | Yes | No |
|  | …you were bothered more than you expected by feelings of grief, or you had frequent intense pangs of grief? | Yes | No |
|  | …you felt that your life had no purpose without the person or thing you lost? | Yes | No |
|  | …grief interfered with your ability to function? | Yes | No |
|  | …your family or friends told you that it was time to get over it? | Yes | No |

# Did you ever have a period of time when you…

|  | …had a great need to reminisce about the person, place or thing you lost? | Yes | No |
| --- | --- | --- | --- |
|  | …spent a lot of time with objects that reminded you of the person, place or thing you lost, such as pictures, scrap books, mementos, etc.? | Yes | No |
|  | …felt compelled to visit places that reminded you of the person, place or thing you lost? | Yes | No |
|  | …had recurrent upsetting images of the person, place or thing you lost? | Yes | No |
|  | …were extremely sad thinking about how special the person, place or thing was? | Yes | No |
|  | …avoided going to the cemetery, going to the place where the person died, or any other place related to the death? | Yes | No |
|  | …could not remember the things you loved, admired or enjoyed about the person you lost? | Yes | No |
|  | …thought you saw, heard or talked with the person(s) you lost? | Yes | No |
|  | …kept thinking you could have prevented the separation or death? | Yes | No |
|  | …blamed yourself for doing something, or not doing something, that you think might have helped the person(s) you lost? | Yes | No |
|  | …felt that if you stopped grieving you would lose the person(s) forever? | Yes | No |
|  | …felt that it would be wrong if your grief were less intense, as though you were betraying the person(s) you lost? | Yes | No |

**Now I’m going to ask you some questions about how you are now.**

Are you the type of person or have others told you that you…

|  | …enjoy or find satisfaction in taking care of people? | Yes | No |
| --- | --- | --- | --- |
|  | …feel the need to always have someone to take care of (or feel lost or aimless if there isn’t someone to take care of)? | Yes | No |
|  | …find it difficult to ask for help? | Yes | No |
|  | …tend to think that people you are close to will always be there? | Yes | No |
|  | …form very close attachments to people and things? | Yes | No |
|  | …have the feeling that you can’t live without the people close to you? | Yes | No |
|  | …get very upset when you lose things that you are attached to? | Yes | No |

**Domain III. POTENTIALLY TRAUMATIC EVENTS**

***Now I’d like to ask you about some upsetting events that may have happened to you.*** Note to interviewer: The point in asking these questions is simply to identify traumatic events, not to characterize them fully. [^c^ indicates criterion symptoms].

Did any of the following things ever happen to you?

|  | …repeated failure in school or at work? | Yes | No |
| --- | --- | --- | --- |
|  | …repeated severe arguments in your family? | Yes | No |
|  | …being repeatedly teased or harassed? | Yes | No |
|  | …being beaten up or physically threatened? | Yes | No |
|  | …unwanted sexual advances? | Yes | No |
|  | …physical or sexual abuse? ^c^ | Yes | No |
|  | …rape? ^c^ | Yes | No |
|  | …being the object of a lawsuit or disciplinary action? | Yes | No |
|  | …being arrested or indicted for a crime? | Yes | No |
|  | …an event that seriously threatened your well-being, employment, professional status, social standing or financial security? | Yes | No |
|  | …a serious medical illness, surgery, or other distressing medical procedure? ^c^ | Yes | No |
|  | …a serious accident or injury (for example, an automobile accident, or plane crash)? ^c^ | Yes | No |
|  | …a disaster (for example, a hurricane, flood, fire, tornado, earthquake, or explosion)? ^c^ | Yes | No |
|  | …being threatened by criminals or terrorists? ^c^ | Yes | No |
|  | …being a victim of a crime (for example, being robbed, assaulted, or mugged)? ^c^ | Yes | No |
|  | …being in a war zone? ^c^ | Yes | No |
|  | …being imprisoned, kidnapped, tortured, or held hostage? ^c^ | Yes | No |

|  | Are there any other upsetting events that happened to you that I haven’t asked you about? What were they?  *Record here:* | Yes | No |
| --- | --- | --- | --- |
|  | Did you ever witness any upsetting events, like the ones we’ve been talking about, that happened to someone else? ^c^ | Yes | No |
|  | Did you ever hear about any upsetting events like these happening to someone else, so that you felt very affected by them? | Yes | No |
|  | Are there periods of time in your life after the age of 5 about which you can remember absolutely nothing? | Yes | No |

**DOMAIN IV. REACTION TO LOSSES OR UPSETTING EVENTS**

*Note to interviewer: Before proceeding, make a note of the losses and events endorsed by the subject so you can refer to these during the rest of the interview. If the subject has not endorsed any losses or events up to this point, skip to Domain IX (Personal Characteristics).*

Did this event or loss make you feel extremely…

|  | …afraid? | Yes | No |
| --- | --- | --- | --- |
|  | …sad? | Yes | No |
|  | …guilty or ashamed? | Yes | No |
|  | …bitter or angry? | Yes | No |
|  | …hopeless or helpless? | Yes | No |
|  | …horrified or disgusted? | Yes | No |
|  | …physically or emotionally numb or paralyzed? | Yes | No |

At the time of the loss or event, did you have any of the following…

|  | …pounding heart, sweating, trembling, or shaking? | Yes | No |
| --- | --- | --- | --- |
|  | …sensations of shortness of breath or choking? | Yes | No |
|  | …chest discomfort or pain? | Yes | No |
|  | …nausea or abdominal distress? | Yes | No |
|  | …feeling dizzy, unsteady, lightheaded, or faint? | Yes | No |

***At the time of the loss or event, did you feel…***

|  | ...like the event wasn’t real, or as if you were in a dream or like you were a spectator? | Yes | No |
| --- | --- | --- | --- |
|  | ...you were doing things automatically, without thinking about them? | Yes | No |
|  | ...your sense of time changed, for example, things seemed to be happening in slow motion? | Yes | No |
|  | ...confused or uncertain about where you were or what time it was? | Yes | No |
|  | ...that colors, sounds and smells were unusually vivid or unbearable? | Yes | No |
|  | ...exceptionally alert or clear-headed? | Yes | No |

**DOMAIN V. Re-experiencing**

Since the loss or event, have you ever…

|  | …had recurrent bad dreams or nightmares about the loss or event, or awakened terrified? | Yes | No |
| --- | --- | --- | --- |
|  | …suddenly gotten bad feelings when you were around certain places, odors, sounds or people? | Yes | No |
|  | …felt or acted as if the events were happening again? | Yes | No |
|  | …had distressing thoughts, feelings, or images related to the loss or event? | Yes | No |
|  | …become more distressed at the time of year when the loss or event occurred? | Yes | No |

|  | Did you notice that other people avoided talking about the loss or event because you got so upset? | Yes | No |
| --- | --- | --- | --- |

***When thinking*** about the loss or event, did you ever…

|  | ...feel upset or have waves of emotion or a sinking feeling in the pit of your stomach? | Yes | No |
| --- | --- | --- | --- |
|  | ...have other physical sensations, such as pain, palpitations, sweating, headache, etc.? | Yes | No |
|  | …feel guilt or shame or blame yourself for what happened? | Yes | No |

**DOMAIN Vi. AVOIDANCE & numbing**

***Did you ever avoid***…

|  | ...thinking or talking about the loss or event? | Yes | No |
| --- | --- | --- | --- |
|  | ...specific places, people, or social situations that reminded you of the loss or event? | Yes | No |
|  | …reading the newspaper or watching certain TV programs or movies because they reminded you of the loss or event? | Yes | No |
|  | …activities or things that evoked feelings of loneliness, crying or other distressing emotions related to the loss or event? | Yes | No |

Since the loss or event, did you ever…

|  | …find that you were unable to remember things connected to the loss or event? | Yes | No |
| --- | --- | --- | --- |
|  | …find that certain activities or things became pointless, meaningless or insignificant? | Yes | No |
|  | …feel that you no longer had emotions you used to have, or that your feelings were dulled? | Yes | No |
|  | ...feel cut-off or detached, or like you were different from other people? | Yes | No |
|  | …have difficulty trusting people, either strangers, people in your family, or your friends? | Yes | No |
|  | …feel that you wouldn’t live a long or satisfying life? | Yes | No |
|  | …feel as if your life was changed forever and things would never be the same? | Yes | No |
|  | …feel as if your personality changed? | Yes | No |

# Domain VIi. maladaptive coping

##### Since the loss or event, did you ever…

|  | …stop taking care of yourself, for example, not getting enough rest or not eating right? | Yes | No |
| --- | --- | --- | --- |
|  | …stop taking prescribed medications or fail to follow-up with medical recommendations, such as appointments, diagnostic tests, or a diet? | Yes | No |
|  | … use alcohol or drugs or over-the-counter medications to calm yourself or to relieve emotional or physical pain? | Yes | No |
|  | ...engage in risk-taking behaviors, such as driving fast, promiscuous sex, hanging out in dangerous neighborhoods? | Yes | No |
|  | …wish you hadn’t survived? | Yes | No |
|  | …think about ending your life? | Yes | No |
|  | ...intentionally scratch, cut, burn or hurt yourself? | Yes | No |
|  | …attempt suicide? | Yes | No |

# Domain VIii. Arousal

Since the loss or event, did you ever…

|  | …have trouble concentrating or paying attention, for example, following the story line of a TV program or book or remembering what you had read? | Yes | No |
| --- | --- | --- | --- |
|  | …feel like you just couldn’t relax or let your guard down? | Yes | No |
|  | …startle easily at the sound of sudden noises, or when someone touched you, spoke to you, or approached you unexpectedly? | Yes | No |
|  | …feel more irritable, have outbursts of anger or rage, or lose your temper over minor things? | Yes | No |
|  | …have more difficulty falling asleep or staying asleep than before or need a light on to go to sleep? | Yes | No |

**DOMAIN IX. Personal Characteristics/Risk Factors**

**Now I’m going to ask you some questions about how you are now.**

Are you the type of person or have others told you that you…

|  | …are extremely sensitive to stress or loss? | Yes | No |
| --- | --- | --- | --- |
|  | …are provocative? | Yes | No |
|  | …like being the center of attention? | Yes | No |
|  | …often follow your instinct without really thinking about what you are doing? | Yes | No |
|  | …usually find exciting what others would find frightening? | Yes | No |
|  | …often engage in reckless or dangerous activities? | Yes | No |
